# Prognostic factors for clinical outcomes after rotator cuff repair

**DOI:** 10.1590/1413-78522015230300992

**Published:** 2015

**Authors:** José Otávio Reggi Pécora, Eduardo Angeli Malavolta, Jorge Henrique Assunção, Mauro Emílio Conforto Gracitelli, João Paulo Sobreiro Martins, Arnaldo Amado Ferreira

**Affiliations:** 1 Institute of Orthopedics and Traumatology, Hospital das Clínicas da Faculdade de Medicina da Universidade de São Paulo, São Paulo, SP, Brazil

**Keywords:** Rotator cuff, Arthroscopy, Prognosis, Evaluation of results of therapeutic interventions

## Abstract

**OBJECTIVE:**

To identify prognostic factors of postoperative functional outcomes.

**METHODS:**

Retrospective case series evaluating patients undergoing rotator cuff repair, analyzed by the UCLA score (pre and 12-month postoperative) and Magnetic Resonance Imaging (preoperative). Patients' intrinsic variables related to the injury and intervention were evaluated. Multivariate linear regression analysis was performed to determine variables impact on postoperative functional assessment.

**RESULTS:**

131 patients were included. The mean UCLA score increased from 13.17 ± 3.77 to 28.73 ± 6.09 (p<0,001). We obtained 65.7% of good and excellent results. Age (r= 0.232, p= 0.004) and reparability of posterosuperior injuries (r= 0.151, p= 0.043) correlated with the functional assessment at 12 months. After multivariate linear regression analysis, only age was associated (p = 0.008).

**CONCLUSIONS:**

The surgical treatment of rotator cuff tears lead to good and excellent results in 65.6% of patients. Age was an independent predictor factor with better clinical outcomes by UCLA score in older patients*. Level of Evidence IV, Case Series.*

## INTRODUCTION

The tendinopathies of the rotator cuff are the leading cause of shoulder pain^1 ^and tears of these tendons affect 20.7% of the population.^2^ The increasing number of surgical repair of these injuries^3^ generates high costs to the health systems.^4^ The arthroscopic repair of the rotator cuff leads to satisfactory clinical outcomes in up to 93% of pacients.^5^^,^ 6 However, the rate of tear recurrence ranges from 4 to 94%.^7^^,^ 8

Several intrinsic factors of the patient, injury or surgery have been associated with the outcomes of treatment of the rotator cuff injuries.^9^^-^ 14 Determining prognostic factors helps identifying patients at increased risk of unsatisfactory development, and elaborate strategies that reduce this occurrence.

Several studies^11^^-^ 13 ^,^ 15 seek correlations isolated and fragmentedly.^16^ Few studies performed independently, through a multivariate analysis, search for predictors of rotator cuff surgical treatment outcomes.^9^^,^ 17 It is known that this form of analysis reduces the confounding factors in the evaluation of different prognostic criteria.^18^

The primary objective of this study was to identify which prognostic factors independently correlate with clinical outcome of patients undergoing repair of the rotator cuff. As a secondary objective we evaluated the clinical outcomes of the procedure. 

## METHODS

A retrospective case series was performed, evaluating patients undergoing rotator cuff repair arthroscopically. The procedures were performed by five different surgeons, which are part of the Shoulder and Elbow Group at our institution. The surgeries took place between June 2006 and April 2013. This study was approved by the Ethics Committee of our institution under the number 696.

Our analysis did not include patients with glenohumeral instability, glenohumeral osteoarthritis, adhesive capsulitis, inflammatory arthropathy or those who did not have preoperative functional assessment and at 12 months follow-up.

In the preoperative period, patients were clinically evaluated by the method of the University of California at Los Angeles (UCLA) and magnetic resonance imaging (MRI). In the postoperative evaluation, the UCLA scale was applied at 12 months of follow-up, as well as Ellmann's categorized assessment.^19^

The following variables were recorded:

1) Intrinsic to the patient: gender, age and affected side.

2) Related to the injury: type of acromion,^20^ affected tendon, injury type (partial or full thickness), and DeOrio and Cofield^21^ rating for posterior superior tears and Lafosse *et al*.^22^ classification for the subscapularis tears;

3) Related to the intervention: procedure carried out in posterior superior portion of the rotator cuff (supraspinatus and/or infraspinatus) and subscapularis (none, debridement, partial or total repair), on the long head of the biceps (none, tenotomy or tenodesis), on the distal portion of the clavicle (none, resection of the inferior osteophytes or Mumford procedure) and acromioplasty.

The procedures were performed under general anesthesia associated with interscalene block. Antimicrobial prophylaxis with first-generation cephalosporin for 24 hours was used. Patients were placed in beach chair and conventional portals (posterior, anterior and lateral) were used. When necessary, additional portals were performed.

Bursectomy was performed in all cases. Acromioplasty was performed in cases where there was tear involving the supraspinatus, provided that it was completely repairable and was not part of an massive rupture. The procedure in the biceps tendon was done in cases with partial tears or instability. Tenotomy was chosen in patients over 60 years old and tenodesis in younger patients. Resection of the distal clavicle portion was indicated when there was pain symptoms associated with acromioclavicular joint disease. The suture of the rotator cuff was performed after debridement of the greater tuberosity, using simple row of anchors and ungrounded points.

Postoperatively, patients were maintained on a sling for six weeks. Active movements to the elbow, wrist and fingers were started on the first day. Passive motion to the shoulder was initiated after three weeks, allowing up to 90° flexion, external rotation and abduction as tolerable. After six weeks, assisted active and free movements in all planes were performed. Strengthening was started after three months.

### Statistical analysis

Continuous variables were evaluated for their normality using the Kolmogorov-Smirnov test and homogeneity, through the Levene test.

The non-parametric Wilcoxon test was used to analyze the impact of surgical treatment on the functional UCLA scale, comparing the preoperative scores to those at 12 months postoperatively. The Pearson correlation coefficients were calculated to determine the correlation between postoperative scores and intrinsic variables to the patient, the injury and the intervention. For continuous or ordinal variables, a positive correlation indicates that as the value of the variable increases, the post-operative score is also higher.

A negative correlation indicates the opposite. For dichotomous variables, a positive correlation indicates that when the variable is present, the postoperative score is higher. A negative correlation indicates the opposite. Regarding gender, a positive correlation indicates that females have higher postoperative score. A negative correlation indicates better results in males, instead.

We performed a multivariate linear regression in order to define the independent variables influencing clinical outcomes at the end of follow-up.

For data analysis we used the SPSS software version 20.0, adopting a significance level of 5%.

## RESULTS

In the study period, 491 patients with pathologies of the rotator cuff were operated at our institution. After selection by the inclusion and non-inclusion criteria, our study included 131 patients (131 shoulders). Of these, 80 (61.1%) were female and 99 (75.6%) had the right side operated. The mean age was 54.97 ± 8.79 years old.

Variables related to the injury, defined by the preoperative imaging studies, are shown in Table 1. The variables related to intervention were detailed in Table 2.

**Table 1.** Variables related to the injury, defined on pre and postoperative imaging tests.

Variables analyzed	n	%
Acromion type		
Flat	10	7.6
Curved	93	71.0
hooked	28	21.4
Tear of the supraspinatus tendon		
Partial	7	5.3
Small	25	19.1
Medium	73	55.7
Large	22	16.8
massive	4	3.1
Tear of the subscapularis tendon		
Absent or partial articular	77	58.8
Complete lesion of superior 1/3	48	36.6
Complete lesion of superior 2/3 or the whole extension	6	4.6

**Table 2.** Variables related to the intervention.

Variables analyzed	n	%
Posterior superior repair		
Not performed	4	3.1
debridement	8	6.1
Partial repair	6	4.6
Complete repair	113	86.3
Subscapular repair		
Not performed	79	60.3
debridement	34	26.0
Partial repair	4	3.1
Complete repair	14	10.7
Procedure on biceps		
None	79	60.3
Tenotomy	13	9.9
Tenodesis	39	29.8
Lateral resection of the clavicle		
Yes	30	22.9
No	101	77.1
Acromioplasty		
Yes	118	90.1
No	13	9.9

According to the UCLA classification, the average score was 13.17 ± 3.77 in the preoperative evaluation and 28.73 ± 6.09 at 12 months of follow-up, showing significant improvement (p <0.001). We obtained 47 (35.9%) patients with excellent results, 39 (29.8%) with good results, and 45 (34.3%) had unsatisfactory outcomes, according to Ellman's classification.

The correlations between the variables intrinsic to the patient, related to the injury and to the intervention are shown in

Table 3. The only variables that correlated with the functional outcome at 12 months postoperative were age (r=0.232, p=0.004) and performed procedure in the posterior superior rupture (r=0.151, p=0.043).

**Table 3.** Univariate correlations between the variables analyzed and the functional outcome by the UCLA scale at 12 months.

Variable	Absolute correlation (r)	*p*
Gender	-0.041	0.32
Age^1^	0.232	0.004 *
Acromion type^1^	-0.095	0.141
Length of the posterior superior tear^1^	0.07	0.212
Length of the subscapular tear^1^	-0.005	0.476
Procedure on the posterior superior tear^1^	0.151	0.043 *
Procedure on the subscapular^1^	-0.01	0.453
Procedure on the biceps^2^	-0.08	0.183
Lateral resection of the clavicle^2^	-0.005	0.476
Acromioplasty^2^	0.069	0.216

1 : Continuous or ordinal variables;

2 : Dichotomous variables.

According to multivariate linear regression, from all variables, only age was independently correlated with the functional evaluation at 12 months (p=0.008). From the data obtained, we can expect a higher score in 0.161 points (range 0.043 - 0.242, 95% CI) by the UCLA scale to each additional patient's year of age. 

## DISCUSSION

Our series included 131 patients, a significant number for the national literature. Checchia et al.^5^ and Godinho et al.^6^ described the results of 141 and 100 patients, respectively. Like other authors, our results showed significant improvement with the procedure. According to Ellmann's classification, we obtained 65.7% of excellent or good results, values similar to those of Godinho et al.^6^ (67%), but lower than those of Checchia et al.^5^ (93.7%). It is worth noting that we performed clinical assessments in a standardized way at 12 months, unlike others (average follow-up over 24 months in both cases). With longer follow-up one could expect an increased score.^23^

McElvany et al.^16^ performed a meta-analysis evaluating the functional outcomes of the rotator cuff repair. In 90 studies, the score obtained in the postoperative period was on average 103% (range 21-272%) higher than preoperatively, results close to the 118% improvement obtained by us. The authors also observed by evaluating 102 studies that the average score after surgery is 85% (range 64-97%) of the maximum allowed by the scales. In our study, the average score represented 82% of the maximum allowed by the UCLA scale.

This study demonstrated that only the patient's age correlated independently with clinical outcomes in multivariate regression, in a positive way. This is in contrary to some authors,^9^^,^ 24 describing worst rating by the functional scales in older patients. However, other autores^13^^,^ 25 observe worse clinical outcomes and lower satisfaction in younger patients. Possible explanations for best results with increasing age may be less functional demand and lower incidence of labor issues. In addition, the UCLA scale, used as a measure of outcome, does not evaluate strength accurately, but categorized.^26^ Thus, the results may not apply to analyzes by other scales and this may be an explanation for the best results having occurred in the older population.

We also observed that the univariate correlation showed that the posterior superior rupture repair procedure also influenced the clinical outcome, with better results in cases with complete repair of the injury. Similarly, O'Holleran et al.^27^ showed that patients with not repairable extensive ruptures and sudmitted only to surgical debridement had worse functional outcomes.

Some authors showed correlation of the dimension of the rupture with clinical outcomes, with larger injuries leading to lesser satisfaction,^27^ more tear recurrences^17^ and worst functional outcomes.^9^^,^ 11 ^,^ 12 Our data did not show similar findings. However, the small number of patients with extensive ruptures limits our conclusions regarding this variable.

There is no consensus regarding the influence of gender in the final outcome. Better clinical outcomes both in men^9^^,^ 11 ^,^ 12 ^,^ 16 as in women^13^ are described. In our study, as in Gulotta's et al.^17 ^and Vastamäki's et al.,^10^ there was no effect of this variable in the postoperative clinical outcomes .

The performance of tenotomy or tenodesis of the long head of the biceps, and the resection of the distal end of the clavicle also did not affect clinical outcomes. These results are in agreement with other authors^9^^,^ 11 ^,^ 12 and indicate that these procedures associated to rotator cuff repair have little influence on functional outcome. We had in our sample a small number of patients not undergoing acromioplasty, which limits our findings on this not being a predictor. Nevertheless, current evidence indicates that holding the anterior inferior resection of the acromion does not change the clinical outcomes.^28^

It was not possible to assess other variables that could influence the results, as the degree of fatty degeneration of the muscles,^10^^,^ 14 the activity level of patients,^10^ symptomatology span,^10^ and labor issues,^13^ the latter being a limitation of our study. The absence of postoperative imaging studies in our analysis is also open to criticism. It is worth noting that failure to detect correlations among the other variables may be due to a Type II error, determined by an insufficient sample.

As a favorable point of our study we can mention the large series according to national standards, our knowledge being of only one article evaluating a higher number of patients with rotator cuff disorders.^5^ Moreover, conducting a multivariate analysis allows the control and evaluation of different prognostic criteria, reducing confounding factors when compared to an univariate analysis.^18^ Our results come contribute for the understanding of the prognostic factors of treatment of these injuries, a field with still no consensus in the orthopedic literature. We believe that further studies are needed, including larger databases and eventually multicenter collaboration, to provide more concrete answers. 

## CONCLUSION

The surgical treatment of rotator cuff tears led to good and excellent results in 65.6% of patients. Age was an independent predictor, with better clinical outcomes by the UCLA scale in elderly patients.
